# Indirubin alleviates retinal neurodegeneration through the regulation of PI3K/AKT signaling

**DOI:** 10.7555/JBR.37.20230078

**Published:** 2024-02-23

**Authors:** Huan Li, Huiying Zhang, Lushu Chen, Yaming Shen, Yuan Cao, Xiumiao Li, Jin Yao

**Affiliations:** 1 Department of Ophthalmology, the Affiliated Eye Hospital of Nanjing Medical University, Nanjing, Jiangsu 210029, China; 2 Department of Ophthalmology, the Fourth School of Clinical Medicine, Nanjing Medical University, Nanjing, Jiangsu 210029, China

**Keywords:** retinal neurodegenerative disease, oxidative stress, PI3K/AKT, retinal ganglion cell, apoptosis

## Abstract

Retinal neurodegenerative disease is a leading cause of blindness among the elderly in developed countries, including glaucoma, diabetic retinopathy, traumatic optic neuropathy and optic neuritis, *etc*. The current clinical treatment is not very effective. We investigated indirubin, one of the main bioactive components of the traditional Chinese medicine Danggui Longhui Pill, in the present study for its role in retinal neurodegeneration. Indirubin exhibited no detectable tissue toxicity *in vivo* or cytotoxicity *in vitro*. Moreover, indirubin improved visual function and ameliorated retinal neurodegeneration in mice after optic nerve crush injury *in vivo*. Furthermore, indirubin reduced the apoptosis of retinal ganglion cells induced by oxidative stress *in vitro*. In addition, indirubin significantly suppressed the increased production of intracellular reactive oxygen species and the decreased activity of superoxide dismutase induced by oxidative stress. Mechanically, indirubin played a neuroprotective role by regulating the PI3K/AKT/BAD/BCL-2 signaling. In conclusion, indirubin protected retinal ganglion cells from oxidative damage and alleviated retinal neurodegeneration induced by optic nerve crush injury. The present study provides a potential therapeutic medicine for retinal neurodegenerative diseases.

## Introduction

Retinal neurodegenerative disease is the most common cause of irreversible blindness worldwide, encompassing conditions such as diabetic retinopathy (DR)^[1]^, glaucoma^[2]^, traumatic optic neuropathy and optic neuritis^[3]^. Many factors are associated with retinal neurodegeneration, including the disruption of neurotrophic factors, oxidative stress, hypoxia/ischemia, mitochondrial dysfunction, inflammation, glutamate excitatory toxicity, and calcium overload^[4]^. These factors activate several molecular pathways to induce retinal ganglion cell (RGC) dysfunction and loss, eventually leading to low vision or blindness.

Several treatment or prevention methods have been proposed to protect RGC function and restore visual function. Current neuroprotective therapies for retinal neurodegenerative disease mainly include antioxidants, glutamate receptor antagonists, calcium channel blockers, and neurotrophic factors^[4]^. However, the clinical treatment effects are often unsatisfactory. Therefore, it is necessary to find a new treatment for retinal neurodegenerative disease.

Indirubin, the main effective ingredient of the Danggui Longhui Pill that is composed of a variety of traditional Chinese medicines. Indirubin is extracted from the roots and leaves of *Isatis indigotica* Fortune. Indirubin is also found in mollusks, human urine, and various bacteria^[5]^. Indirubin is known to be used for the treatment of leukemia^[6]^. Furthermore, indirubin plays a crucial role in anti-inflammation^[7]^, anti-oxidation^[8]^, inhibition of angiogenesis^[9]^, and neuroprotection^[10]^. Several studies have reported that oxidative stress may induce RGC apoptosis by activating mitochondrial pathways^[11]^. It is necessary to search for a method to inhibit the RGC apoptosis induced by oxidative stress, which may provide an effective treatment for retinal neurodegenerative diseases. Because indirubin has been reported to be used for treating various neurodegenerative conditions, we speculated that it might be used for the treatment of retinal neurodegenerative diseases.

In the present study, we used optic nerve crush (ONC) injury and RGC damage induced by H_2_O_2_ to simulate disease processes to investigate whether indirubin serves as a potential medicine for the treatment of retinal neurodegenerative diseases.

## Materials and methods

### Animals

All male C57BL/6J mice (six to eight weeks of age) were purchased from the Nanjing Qinglongshan Experimental Animal Center (Nanjing, Jiangsu, China) and raised in a standard pathogen-free environment with a temperature of 22 (± 2) ℃, humidity of 50% (± 5%), and maintained on a 12 h light/dark cycle with free access to food and water. The mice were randomly divided into four groups: a normal control group, an ONC group, an ONC + 0.9% Saline group, and an ONC + Indirubin group, with 20 mice in each group.

All the animal experimental procedures followed the protocols approved by the Institutional Animal Care and Use Committee at Nanjing Medical University and adhered to the Association for Research in Vision and Ophthalmology (ARVO) Statement for the Use of Animals in Ophthalmic and Vision Research.

### Intravitreal injection

Prior to subretinal injections, mice were topically administered 0.5% proparacaine, 1% tropicamide, and 2.5% phenylephrine, and anesthetized with ketamine (100 mg/kg body weight) and xylazine (10 mg/kg body weight). To initiate the injection, we placed 2.5% hypromellose over the eye, and used a 30-gauge needle to make an incision in the limbus. A glass coverslip was then placed over the eye to allow for visualization of the retina. Going through the scleral incision in the limbus, and using a Hamilton syringe with a 33-gauge blunt needle, we delivered 1 μL of PBS or indirubin (10 μmol/L; Cat. #HY-N0117, MCE, Shanghai, China) to the subretinal space.

### ONC model

Mice (six to eight weeks old) were sedated by subcutaneous injection of ketamine/xylazine solution at 1 g/μL body weight. We used surgical forceps to expose the optic nerve and crush it for 10 s at a distance of approximately 2 mm from the posterior of the eyeball. We carefully avoided damaging the ophthalmic artery. Antibiotic ointment was applied postoperatively to protect the cornea from infection.

### Flash visual evoked potentials (F-VEP) recording

Seven days after the ONC injury, full-field flash electroretinography was used to assess retinal function. After the mice were anesthetized, we inserted subcutaneous platinum needle electrodes into the skin in the middle of both ears. The needle electrodes at the bottom of the cheekbag and the tail were used as a reference electrode and a grounding electrode, respectively. Background illumination was turned off with a flash intensity of 3 cd·s^–1^·m^–2^ and a single flash with a flash rate of 1.30 Hz as well as a test average of 64 sweeps. VEP responses were measured by the Espion Visual Test System (Diagnosys LLC, Lowell, MA, USA).

### Electroretinogram (ERG) recording

ERGs were used to assess the safety of indirubin. After the mice were anesthetized, we placed a subcutaneous platinum needle electrode on the surface of the cornea. The needle electrodes at the bottom of the cheekbag and tail were used as the reference electrode and the grounding electrode, respectively. Background illumination was turned off with a flash intensity of 4 cd·s^–1^·m^–2^. ERG responses were measured by the Espion Visual Test System (Diagnosys LLC).

### Hematoxylin and eosin (HE) staining

Seven days after ONC, retinas were dissected from the mouse eyes that were fixed in 4% paraformaldehyde (Cat. #BL539A, Biosharp, Shanghai, China) for 24 h. Subsequently, samples were sectioned at a thickness of 5 µm in paraffin. Only the central part of each retina was selected as the slice object. The sections were stained with hematoxylin and eosin (Cat. #BP-DL001, Sbjbio, Nanjing, Jiangsu, China). The images were taken under a light microscope (IX73-TH4-200, Olympus, Tokyo, Japan). The ganglion cell layer in the central part of the retina was selected for counting.

### Terminal deoxynucleotidyl transferase-mediated dUTP-biotin nick end labeling (TUNEL) staining

The detection of RGC apoptosis was conducted by the TUNEL assay kit (Cat. #C1091, Beyotime, Shanghai, China) according to the manufacturer's instructions. TUNEL staining was performed with fluorescein-dUTP to stain apoptotic cell nuclei, and DAPI (5 mg/mL) was used to stain all cell nuclei at 37 ℃ for 5 min. The cells in which the nucleus was stained with fluorescein-dUTP were defined as TUNEL-positive cells. The slides were visualized under a fluorescence microscope (IX73-TH4-200).

### Immunofluorescence staining

Enucleated eyes were fixed in 4% paraformaldehyde at 4 ℃ overnight. Eyes were incubated with 30% sucrose for 48 h and then quickly frozen in optimal cutting temperature compound mounting media (Cat. #4583, Sakura, Saint Joseph, USA). Retinal cross sections (12 μm) were cut at −20 ℃. The antibodies anti-NeuN (1∶400 dilution; Cat. #36662, Cell Signaling Technology, Danvers, MA, USA) and anti-TUBB3 (1∶500 dilution; Cat. # ab18207, Abcam, Cambridge, UK) were used for immunofluorescence staining. Sections were incubated with fluorophore-conjugated secondary antibody (1∶1000 dilution; Cat. #7076, Cell Signaling Technology) at room temperature in the dark for 2 h. The retinal sections were counterstained with DAPI for 10 min. Representative images were observed with a fluorescence microscope (IX73-TH4-200). The fluorescence intensity was analyzed by Image J.

### Visual cliff test

The visual cliff test apparatus consists of an open-top plexiglass box, with dimensions of 60 cm × 60 cm × 30 cm, separated by a center platform (3 cm high and 5 cm wide) into two regions, a shallow side with a checkerboard pattern immediately under it, and a deep side with the same checkerboard pattern placed at a depth of 30 cm to create the illusion of depth. Mice were placed onto the center platform, and their choices to step down were recorded. Each mouse was subjected to the test once. The box and central platform were thoroughly cleaned after each test.

### Looming visual stimulus response test

The test for the looming visual stimulus response was performed in a device with dimensions of 30 cm × 50 cm × 30 cm. An 8 cm wide board was placed at one end of the device at a height of 8 cm to serve as a hideout. To encourage mice to explore their environment and remain outside of the hideout, food was placed on the opposite side of the hideout. A monitor was placed on top of the device to display the looming stimulus, a video of an expanding black disk on a gray background created using software. The stimulus parameters consisted of a circle expanding from a radius of 2 degrees to 20 degrees in 250 ms, where it remained for 250 ms. The stimulus was displayed 15 times, with a 500 ms interval between presentations. A camera was used to record mouse behavior. Mice were placed in the device for 10 min prior to the stimulus onset to adapt to the device. The responses were assessed during the looming stimulus. If a mouse attempted to find a hideout to escape the stimulus, it was considered a positive looming responder. Each mouse underwent the test once. The device was thoroughly cleaned after each test.

### Extraction and purification of primary RGCs

Twelve retinas dissected from two-day-old C57BL/6 mice were incubated in Hank's Balanced Salt Solution (HBSS) containing 0.2 mg/mL N-acetyl-L-cysteine, 16 units/mL papain, and 0.04% DNase at 37 ℃ for 20 min. Tissues were shaken in HBSS containing 0.15% trypsin inhibitor and 0.12% bovine serum albumin for 30 s.

Retinal cell suspensions were washed in HBSS once, resuspended in HBSS containing 4% BSA and incubated with PE-Cyanine7 conjugated CD90.2 (Thy-1.2) antibody (1∶2000 dilution; Cat. #25-0902-81, Thermo Fisher Scientific, Massachusetts, USA) for 20 min to label RGCs for cell sorting. Fluorescence-activated cell sorting was performed with flow cytometry to collect RGCs. Then, the cells were resuspended in a specific culture medium (Cat. #CM-M122, Procell, Wuhan, China) and seeded into a 6-well plate for growth.

### Cell Counting Kit-8 (CCK-8) assay

Following the instruction of the manufacturer, we performed the CCK-8 (BS350-B, Biosharp, China) assay to examine cell viability. RGC cells were seeded into a 96-well plate at 5000 cells/well with 100 μL DMEM and incubated for 24 h. After different treatments, 10 μL of CCK-8 was added to the 96-well plate for 1 h in the dark. The optical density value was measured at 450 nm using a plate reader (Molecular Devices, San Jose, CA, USA)

### Flow cytometry analysis

Annexin V-FITC/PI (Cat. #A211-01, Vazyme, Nanjing, China) was used to measure cell apoptotic rate. After receiving different treatments, RGCs from each group were washed with PBS three times, collected by centrifugation at 1200 *g* for 5 min and resuspended gently in 500 μL of 1× binding buffer. The cells were stained with Annexin V/PI and incubated in the dark for 20 min at room temperature. Next, the samples were immediately analyzed by flow cytometry (Beckman Coulter, Brea, CA, USA).

### Western blotting assay

The proteins were lysed with a cold RIPA buffer and subjected to electrophoresis on a 10% SDS-PAGE gel. The membranes were blocked in 5% dry non-fat milk at room temperature for 2 h and then incubated with primary antibodies at 4 ℃ overnight. The following antibodies against AKT (1∶1000 dilution; Cat. #4695), p-AKT (1∶1000; Cat. #4060), BAD (1∶1000 dilution; Cat. #9268), SOD2 (1∶1000 dilution; Cat. #13141), or the loading control GADPH (1∶1000 dilution; Cat. #5174) purchased from Cell Signaling Technology were used. The blots were then incubated with species-specific HRP-conjugated secondary antibodies (1∶1000 dilution; Cat. #ab6721, Abcam) by gentle shaking at room temperature for 2 h, and the bands were visualized using an enhanced chemiluminescence Western kit (Cat. #BI-WB004, Sbjbio).

### Measurement of intracellular reactive oxygen species (ROS) levels

The ROS Assay Kit (S0033S, Beyotime) was used to measure intracellular ROS levels; 2′,7′-dichlorofluorescein-diacetate (DCFH-DA) was easily oxidized to fluorescent dichlorofluorescein by intracellular ROS. Briefly, the cells were seeded in 96-well plates and treated differently. Following the treatment, the cells were incubated with DCFH-DA at 37 ℃ for 20 min and then observed using a multifunctional enzyme marker (Filter Max F5, Molecular Devices) and measured at 488 nm excitation and 525 nm emission wavelengths, respectively.

### Network pharmacology

The SuperPred database (https://prediction.charite.de/) and the Swiss Target Prediction platform (http://www.swisstargetprediction.ch/) were used to retrieve and predict indirubin targets. Duplicate targets and non-human targets were removed. The protein names were converted to gene names using the UniProt database (https://www.uniprot.org/). Using "Retinal neurodegeneration" as the keyword in DrugBank (https://www.drugbank.ca/), OMIM (https://www.omim.org/), and GeneCards (https://www.genecards.org/) databases to obtain retinal neurodegeneration-related targets, select human-derived targets, and remove duplicate targets after integration. The predicted potential targets of indirubin and retinal neurodegeneration disease-related targets were intersected to obtain common drug-disease targets, which are indirubin's anti-retinal neurodegeneration targets. For the screened common drug-disease targets, the metascape (https://metascape.org/) database was used for GO, including biological process, cellular component and molecular function, and KEGG pathway enrichment analysis.

### Statistical analysis

Data were presented as the means ± standard deviation. Differences between groups were examined using Fisher's exact test or one-way analysis of variance performed by using GraphPad Prism 8 (GraphPad Software, San Diego, CA, USA). A *P*-value of less than 0.05 was considered statistically significant.

## Results

### Indirubin had no detectable tissue toxicity *in vivo* or cytotoxicity *in vitro*

**Figure 1:**
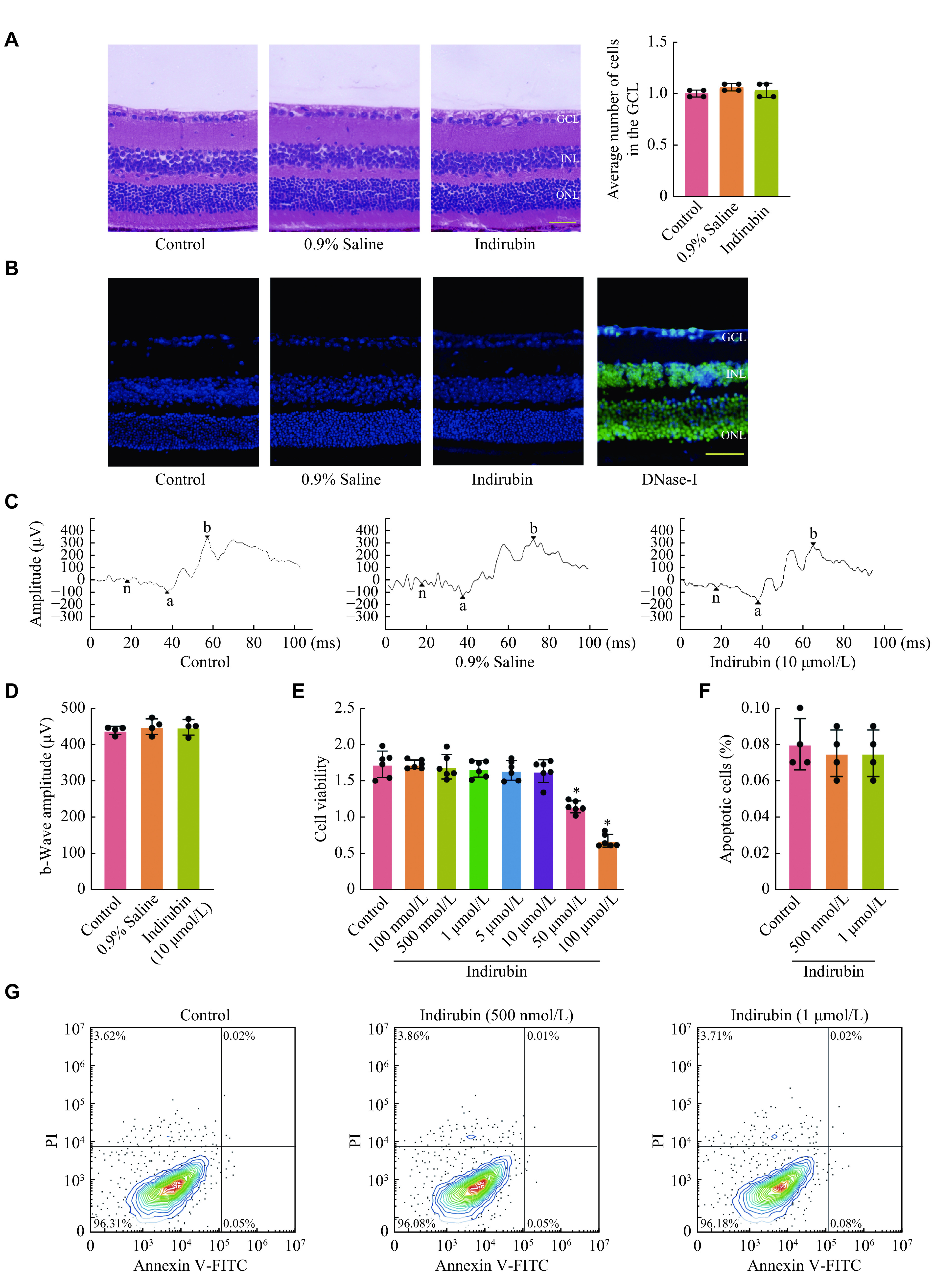
Indirubin had no detectable tissue toxicity *in vivo* or cytotoxicity *in vitro*.

We first evaluated tissue toxicity of indirubin in the retinas of mice. Indirubin or 0.9% saline was injected into the eyes of mice, and the morphological changes in the retinas were detected by HE staining 7 days later. The result showed that there was no morphological change in the retinas of both two groups (***Fig. 1A***). In addition, a similar result was observed by TUNEL staining. No apoptotic cell death in the retina was observed after indirubin administration (***Fig. 1B***). The F-ERG results showed that there was no significant difference between the indirubin (10 μmol/L) group and normal mice groups in the amplitude of b-wave (***Fig. 1C*** and ***1D***). Then, we investigated whether indirubin was cytotoxic in RGCs *in vitro*. The CCK-8 assay showed that indirubin did not affect the viability of RGCs at the concentration of 10 μmol/L or lower (***Fig. 1E***). Annexin V-FITC/PI double labeling assays showed that indirubin (500 nmol/L and 1 μmol/L) did not induce significant cell apoptosis, as shown by less than 5% of the PI-positive RGCs (***Fig. 1F*** and ***1G***). In summary, these results indicate that indirubin has no significant tissue toxicity *in vivo* or cytotoxicity *in vitro*.

### Indirubin alleviated retinal neurodegeneration induced by the ONC injury *in vivo*

Retinal neurodegeneration is the early event in the pathogenesis of DR and glaucoma^[12–13]^. To identify the effect of indirubin on retinal neurodegeneration, the ONC model was used to mimic retinal neurodegeneration.

**Figure 2:**
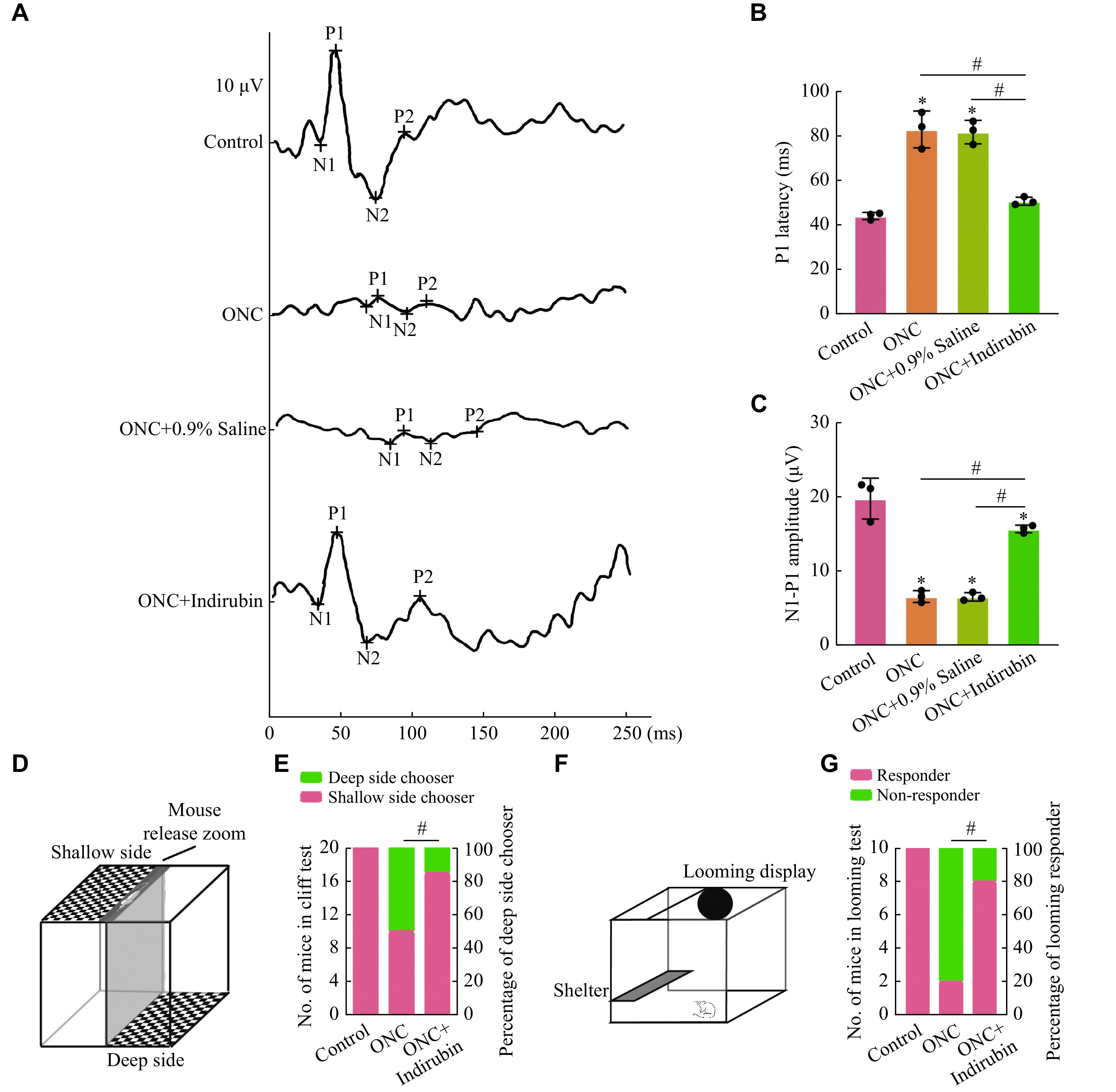
Indirubin alleviated retinal neurodegeneration induced by the ONC injury.

F-VEPs were recorded seven days after ONC induction to investigate whether indirubin could alleviate visual signal conduction disorders induced by the ONC injury. The changes of the F-VEPs in mice of each group were shown (***Fig. 2A***). The latency of P1 wave represents the signal transduction time from the retina to the occipital cortex. On the seventh day after the ONC injury, the latency of P1 wave ([82.83 ± 6.79] ms) was significantly increased in the ONC mice, compared with the control group ([43.83 ± 1.31] ms). It is worth noting that the latency of P1 wave in the indirubin treatment group significantly decreased to 50.50 (± 1.47) ms (***Fig. 2B***), indicating that the response time of mice to light stimulation decreased after indirubin treatment. While the amplitude of N1-P1 (6.50 [± 0.65] μV) significantly reduced in ONC mice, compared with the control group (19.67 [± 2.25] μV). As expected, intravitreal injection of indirubin increased the amplitude of N1-P1 (15.57 [± 0.42] μV) (***Fig. 2C***), indicating that the response ability of mice to light stimulation was improved. These results indicate that indirubin may alleviate visual dysfunction induced by the ONC injury.

Next, a visual cliff test was performed to assess the ability of mice to distinguish visual depth (***Fig. 2D***). This test was based on the instinctive tendency of mice to stay away from dangerous deep side cliffs and prefer to choose safer shallow platforms for visual cliffs. We recorded the direction chosen by the mouse. In the uninjured control group, all mice chose the safer shallow side. In the ONC group, half of the mice chose the shallow side and the other half chose the deep cliff, indicating the randomness of their selection and lack of ability to distinguish visual depth. In contrast, 17 out of 20 mice in the indirubin group chose the shallow side, indicating that indirubin protected the depth perception function of ONC injured mice (***Fig. 2E***).

Finally, we evaluated the innate defense response of mice to approaching hazards (***Fig. 2F***). The experiment was performed in a closed environment. It had an overhead player that could display stimulus signals, and a shelter for mice to hide as well as a camera that can record mouse behavior. For visual stimuli that gradually become larger and closer, mice with normal vision showed a tendency to seek for shelter. Therefore, we recorded that if a mouse made an avoidance response to a gradually approaching dangerous stimulus, it was a positive responder to the stimulus. In the uninjured group, all 10 mice responded, reflecting that the normal mice took evasive measures against imminent danger. After the ONC injury, only three out of 10 mice responded to upcoming stimulus, reflecting that the ability to respond to dangerous stimulus decreased in the ONC group mice. It is worth noting that eight out of the 10 mice treated with indirubin responded to stimulation, reflecting that indirubin could enhance the ability to respond to dangerous stimulus after ONC injury (***Fig. 2G***). These behavioral experimental results indicate the effective role of indirubin in improving visual function in mice with the ONC injury.

**Figure 3:**
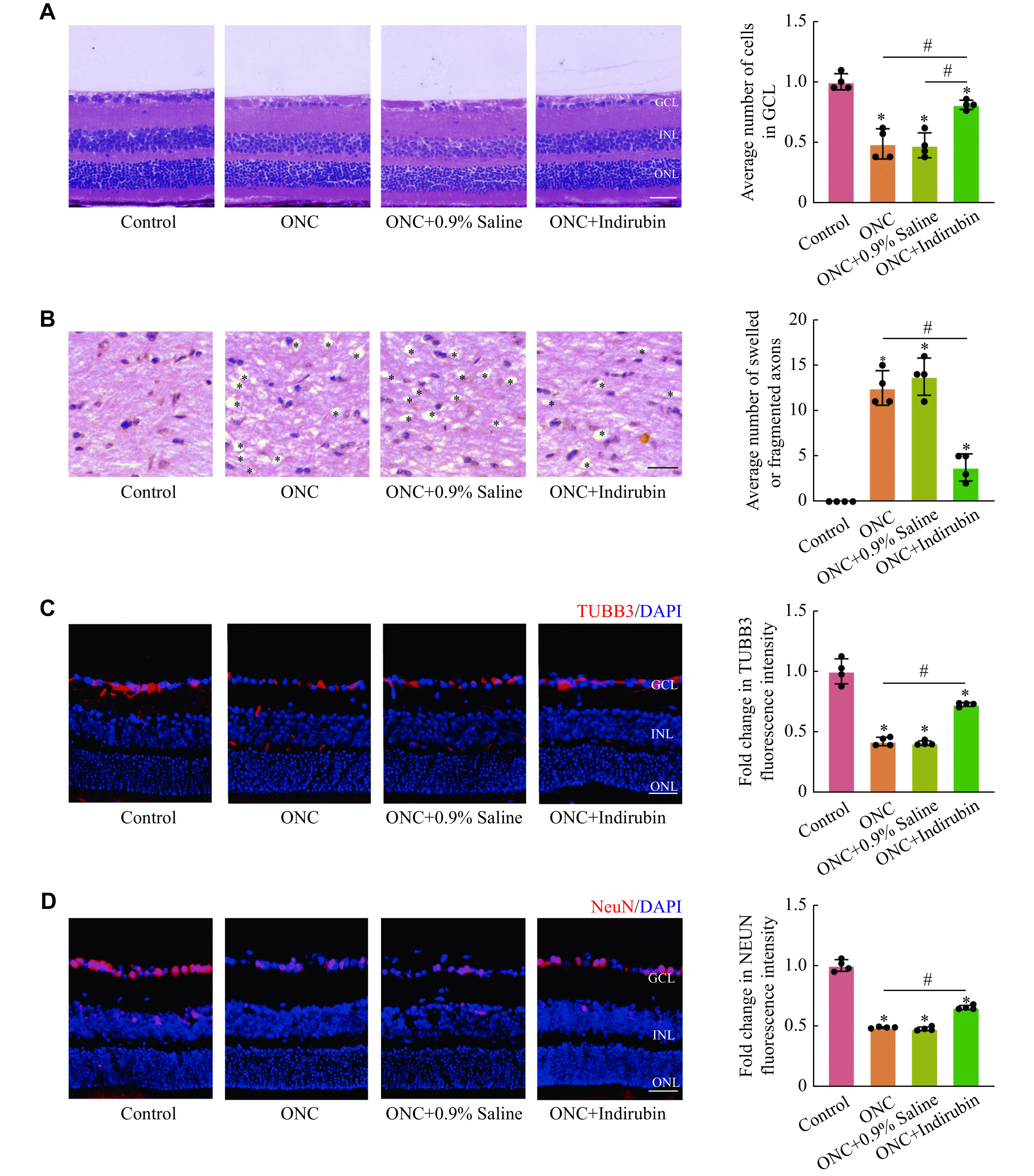
Indirubin promoted the survival of RGCs *in vivo*.

In the following experiment, we evaluated the role of indirubin in the morphological changes of the retina after the ONC damage. HE staining showed that the number of RGCs decreased after the ONC injury, and indirubin suppressed the decrease of RGCs induced by the ONC injury (***Fig. 3A***). HE staining also showed that RGC axons swelled and fragmented after the ONC injury, and the treatment with indirubin alleviated RGC axon injury after the ONC injury (***Fig. 3B***). Moreover, immunofluorescent staining showed that indirubin may have a beneficial effect on facilitating RGC survival, as shown by the increased TUBB3 staining and NeuN staining after the ONC injury (***Fig. 3C*** and ***3D***). Collectively, these results indicate that indirubin may alleviate retinal neurodegeneration induced by the ONC injury.

### Indirubin regulated RGC survival *in vitro*

**Figure 4:**
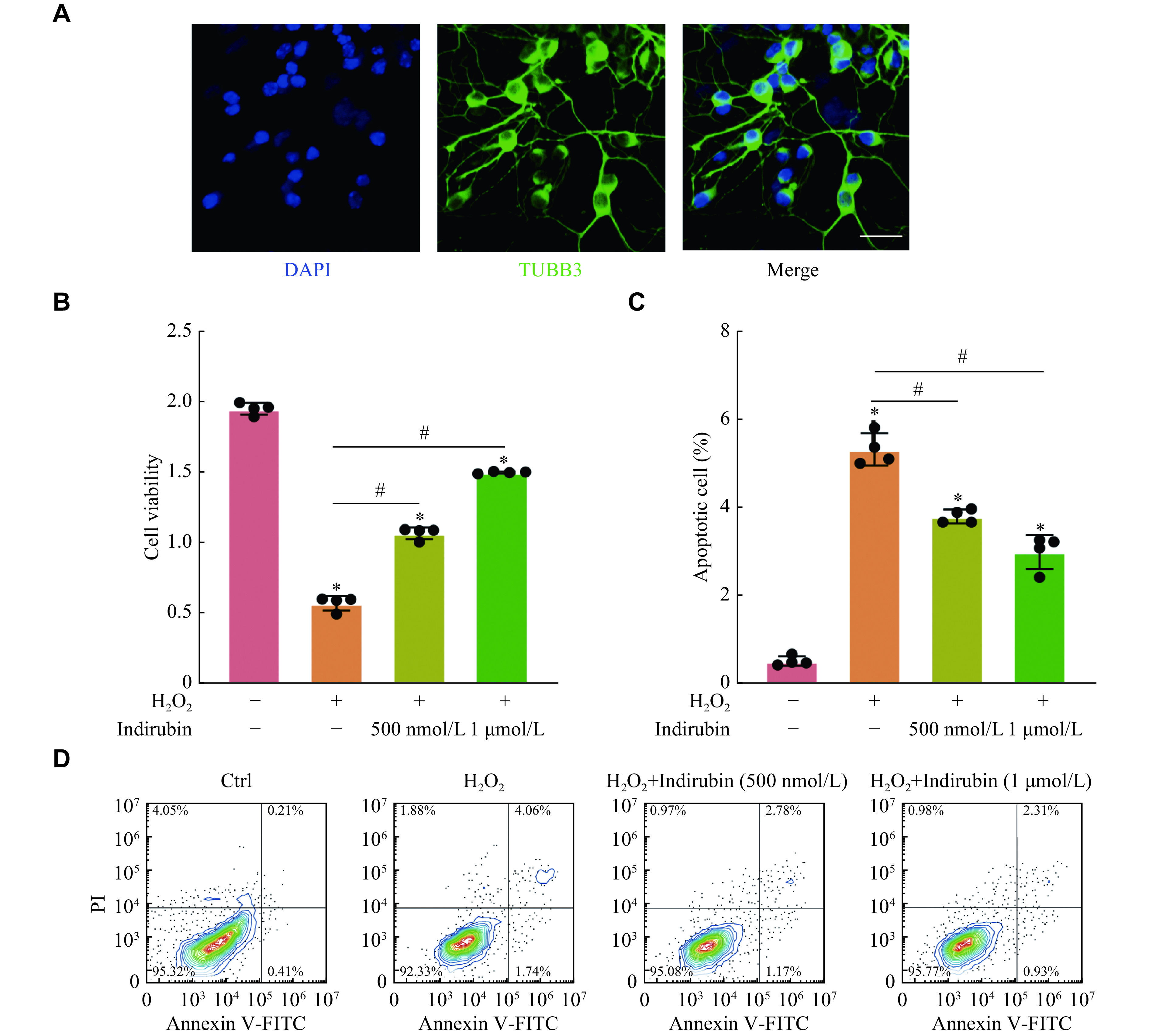
Indirubin regulated RGCs survival *in vitro*.

Based on the above-mentioned results, we speculated that indirubin played a neuroprotective role in the ONC injury-induced retinal neurodegeneration by protecting RGCs. Then, we studied the effect of indirubin on RGC survival *in vitro*. We cultured primary RGCs from the retina of mice and performed TUBB3 immunofluorescence staining to identify whether the cultured cells were RGCs. The results showed that approximately 90% of cells were TUBB3 positive cells, serving as specific markers for central and peripheral neurons (***Fig. 4A***). The cells were used for the subsequent experiments. Then, we used hydrogen peroxide (H_2_O_2_, 200 μmol/L) to stimulate RGCs to mimic oxidative damage. The results of CCK-8 assay showed that H_2_O_2_ treatment significantly reduced the viability of RGCs, and indirubin could increase the viability of RGCs induced by oxidative damage (***Fig. 4B***). Then, we used annexin V-FITC/PI double labeling to determine whether indirubin had a protective effect on RGCs apoptosis induced by oxidative damage. The results showed that indirubin significantly suppressed RGC apoptosis as shown by the decreased PI-positive cells (***Fig. 4C*** and ***4D***). In conclusion, these results indicate that indirubin plays a protective role in the regulation of RGC function *in vitro*.

### Effect of indirubin on ROS formation and antioxidant enzyme activity

**Figure 5:**
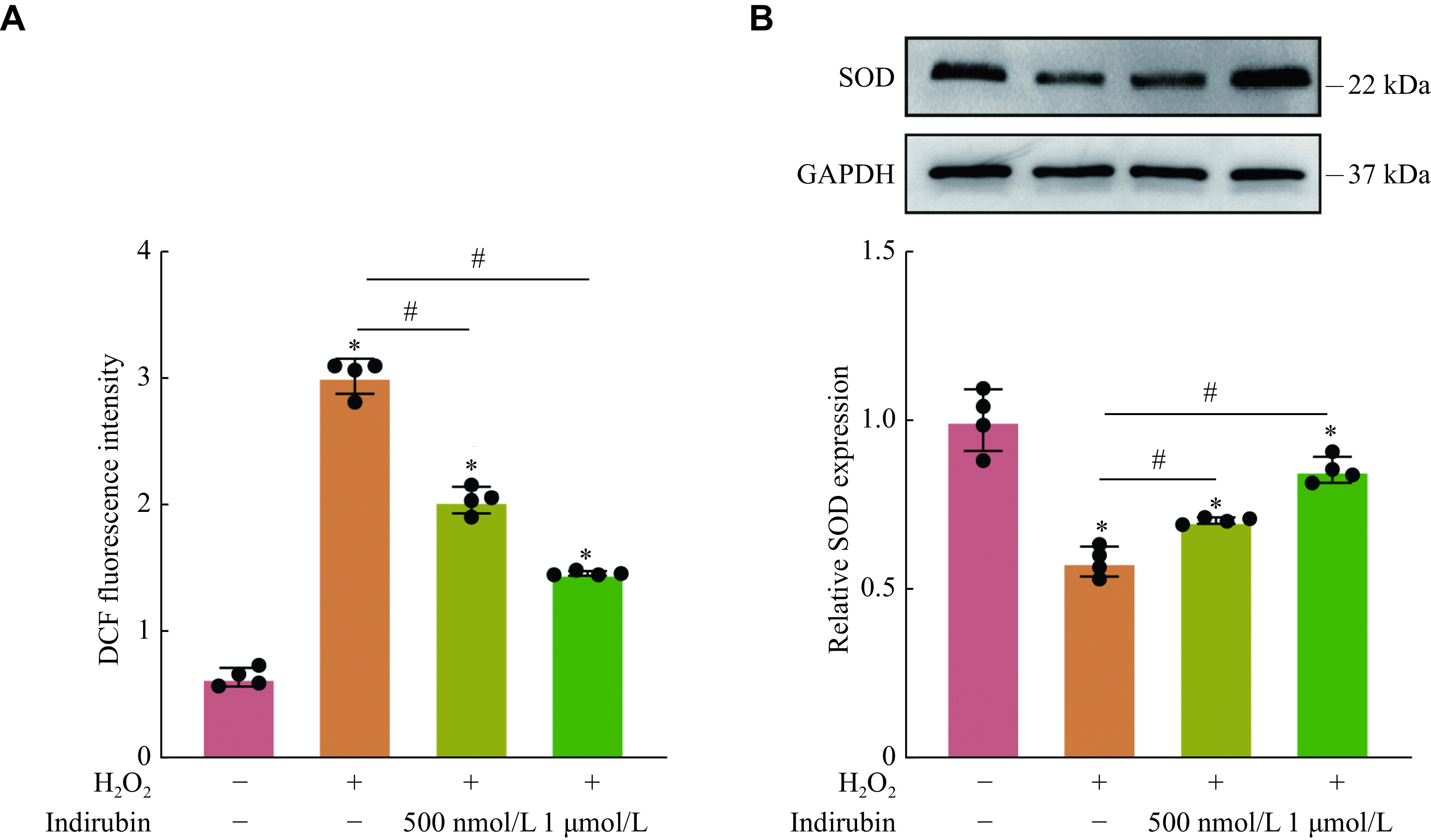
Effect of indirubin on ROS formation and antioxidant enzyme expression.

Oxidative stress is one of the prominent pathogenic factors in retinal neurodegenerative disease, and it is the result of the imbalance between oxidation and antioxidation caused by free radicals^[14]^. ROS is an important factor causing oxidative stress, and its production greater than consumption can cause mitochondrial damage, thereby affecting normal activity of RGCs, leading to RGC apoptosis^[15–16]^. The internal antioxidant enzyme system is very important for scavenging ROS, and SOD2 is one of the most important antioxidant enzymes^[17]^. To further elucidate potential cytoprotective mechanisms of indirubin, intracellular ROS levels and antioxidant enzyme expression were assessed using a DCFH-DA probe and Western blotting, respectively. A higher fluorescent dichlorofluorescein value was observed in the H_2_O_2_-treated group than in the untreated group in RGCs, indicating a significant increase in ROS production (***Fig. 5A***). Indirubin pretreatment significantly decreased the intracellular ROS production. Similarly, the SOD expression level significantly decreased after H_2_O_2_ treatment, while pretreatment with indirubin increased the SOD expression level after oxidative damage (***Fig. 5B***).

### Indirubin played its protective role through the regulation of PI3K/AKT/BAD/BCL-2 signaling pathway

**Figure 6:**
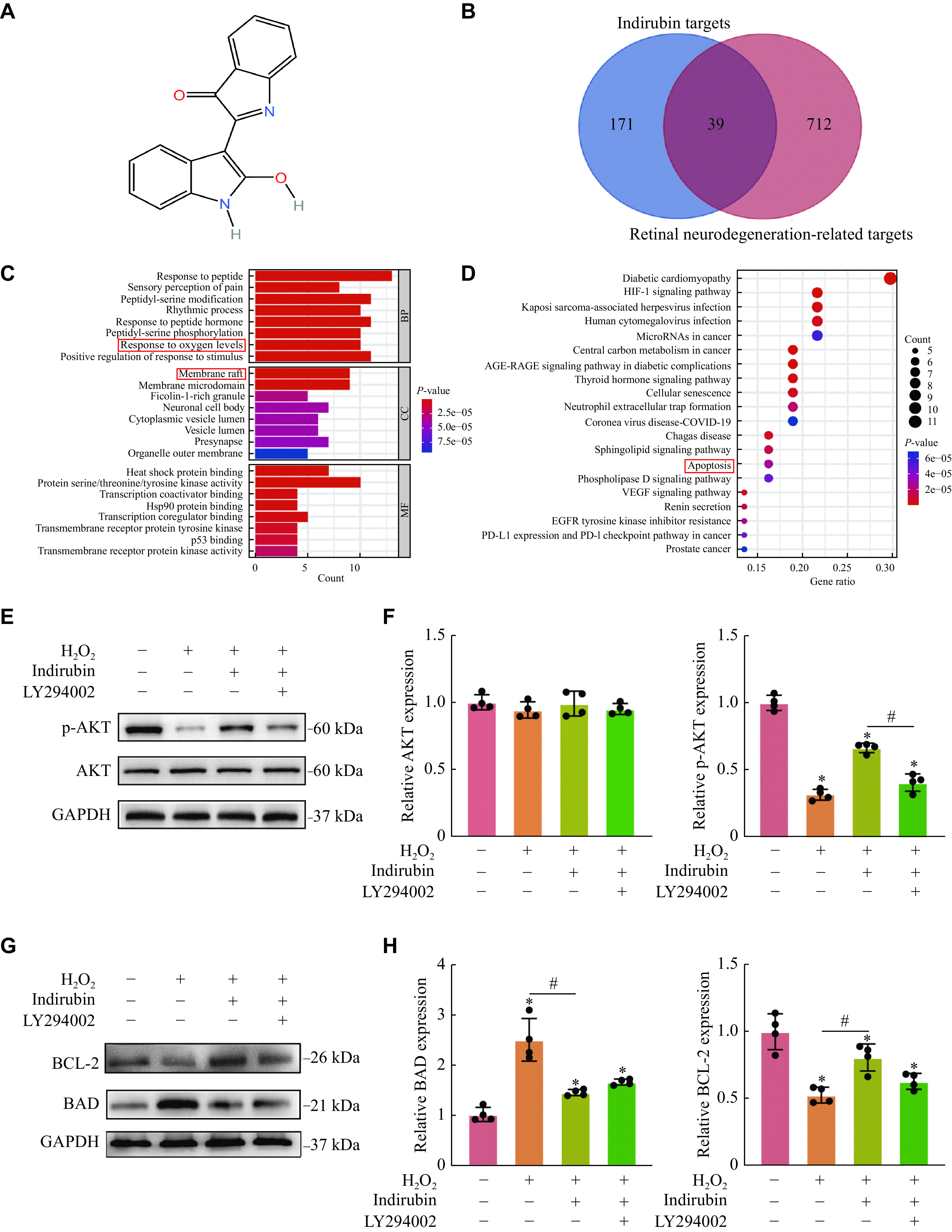
Indirubin played its protective role through the regulation of PI3K/AKT/BAD/BCL-2 signaling pathway.

As shown in ***Fig. 6A***, we predicted the possible pharmacological targets of indirubin based on its structure. We selected the common targets of indirubin and retinal neurodegenerative diseases (***Fig. 6B***). Then, we employed network pharmacology to predict the mechanism by which indirubin played a protective role in retinal neurodegeneration, and predicted that indirubin could play its neuroprotection by reducing the apoptosis of RGCs (***Fig. 6C*** and ***6D***). The PI3K/AKT signal cascade is involved in the pathogenesis and progression of neurodegenerative diseases^[18]^. To study whether indirubin plays its role by regulating the PI3K/AKT signal transduction, we detected the phosphorylation level of AKT in RGCs by Western blotting. As shown in ***Fig. 6E*** and ***6F***, H_2_O_2_ stimulation led to a significant decrease in the level of AKT phosphorylation, while indirubin could increase the level of phosphorylated AKT. However, the effect of indirubin on enhancing the level of phosphorylated AKT was partially suppressed, when cells were preconditioned with the PI3K inhibitor LY294002. These data suggest that indirubin may regulate RGC function by activating the PI3K/AKT signal transduction.

A previous study showed that the PI3K/AKT signal pathway regulated cell apoptosis by modulating the expression of BCL-2 and BAD proteins^[19]^. BCL-2 protein is known to promote cell survival, and BAD protein is known to promote cell apoptosis. Western blotting assays showed that the expression level of BCL-2 decreased and the expression level of BAD increased after H_2_O_2_ exposure, while indirubin increased the expression level of BCL-2 protein and decreased the expression level of BAD protein. However, the effect of indirubin was partially suppressed, when cells were preconditioned with the PI3K inhibitor LY294002 (***Fig. 6G*** and ***6H***). These data indicate that indirubin may play its protective role through the regulation of PI3K/AKT/BAD/BCL-2 signaling pathway.

## Discussion

Retinal neurodegenerative disease is an important cause of blindness in the middle-aged and elderly populations^[1]^. With the acceleration of the aging process, the morbidity of retinal neurodegenerative diseases has gradually increased annually^[20–21]^. Current neuroprotective therapies for retinal neurodegenerative diseases mainly include antioxidants, neurotrophic factors, glutamate receptor antagonists, calcium channel blockers, and anti-inflammatories^[22]^. However, these therapies are not very effective. Therefore, it is necessary to develop new treatment methods for retinal neurodegenerative diseases.

Indirubin is a kind of double indole alkaloid, one of the main bioactive components of a traditional Chinese medicine (Danggui Longhui Pill). Previous studies showed that indirubin was capable of ameliorating neurodegeneration that occurred in animal models of brain diseases^[23–24]^. Indirubin also could make neural progenitor cells acquire tolerance to injury in an oxygen-glucose deprivation hypoxia model *in vitro*^[10]^. Thus, we speculate that indirubin may exert neuroprotective effects in retinal neurodegenerative diseases. In the current study, we identified a role of indirubin in ameliorating retinal neurodegeneration induced by the ONC injury model *in vivo* and the RGC injury induced by H_2_O_2_ *in vitro* without toxicity. These results suggest that indirubin plays a neuroprotective role in retinal neurodegeneration.

Oxidative stress has been considered to play a crucial role in retinal neurodegenerative diseases, including glaucoma^[25]^, DR^[26]^, traumatic optic neuropathy^[3]^, and optic neuritis^[25]^. Under normal physiological conditions, there is a balance between the generation of ROS and the endogenous antioxidant defense systems, and oxidative stress results from the breaking of such a balance. ROS are mainly produced in mitochondria and play a critical role in RGC apoptosis signaling, disrupting the antioxidant capacity of surrounding glial cells^[27]^. SOD is a representative antioxidant enzyme of endogenous antioxidant defense systems that can decompose superoxide into oxygen and hydrogen peroxide. It is known that controlling oxidative stress could prevent the development of retinal neurodegenerative diseases^[28]^. Many therapeutic drugs with neuroprotective effects are mainly correlated with their antioxidant functions^[29]^. It has been reported that the potential neuroprotective effect of vitamins is mainly because of their antioxidant activity^[29–31]^. In the present study, when RGCs were exposed to H_2_O_2_, the antioxidant defense systems were damaged as shown by the increased ROS production and the decreased SOD activity. However, indirubin treatment dramatically increased the activity of the antioxidant enzyme SOD and inhibited the production of ROS. These results suggest that indirubin protects RGCs from intracellular oxidative damage by its antioxidant property.

To investigate the mechanisms underlying the neuroprotective function of indirubin, we analyzed the network pharmacology. The results showed that indirubin could play its neuroprotection by inhibiting the apoptosis of RGCs. The PI3K/AKT signaling has also been shown to be involved in the apoptotic death of neurons^[32]^. For example, Elsherbiny NM *et al*^[33]^ reported that the activating PI3K/AKT signaling could alleviate retinal and optic nerve degeneration in diabetic mice. This is consistent with the present study's results, that is, the phosphorylation levels of Akt markedly reduced after exposure to H_2_O_2_, partly reversing the conditions by indirubin. Furthermore, pre-treatment with the specific PI3K inhibitor LY294002 suppressed the protective effect of indirubin. Therefore, the present study finds that indirubin protects against H_2_O_2_-induced RGC injury through the PI3K/AKT pathway.

Previous studies showed that the PI3K/AKT signal pathway regulated cellular apoptosis by modulating the expression of BCL-2 and BAD proteins^[19]^. BCL-2 is an anti-apoptotic protein that can inhibit apoptosis by restraining cytochrome release. BAD is a pro-apoptotic protein that can enhance apoptosis by contributing to mitochondrial membrane depolarization and permeability. The balance between BCL-2 and BAD has an effect on cellular apoptosis. For example, Ye D *et al*^[19]^ found that the apoptosis of RGCs was reduced by the expression inhibition of BCL-2 antagonistic protein. Lin B *et al*^[34]^ reported that nerve growth factor could protect RGCs by decreasing the level of pro-apoptotic protein Bad and increasing the level of anti-apoptotic protein BCL-2. To further explore the mechanisms of anti-apoptosis modulated by indirubin, we evaluated the expression level of crucial apoptosis-related proteins (BCL-2 and BAD) in RGCs oxidative damage. The results consistently displayed the downregulation of BCL-2 expression and upregulation of Bad expression exposure to H_2_O_2_, which were effectively partially reversed by indirubin. After adding the PI3K inhibitor LY294002, the effect of indirubin was partially inhibited. These results further demonstrate that indirubin may play its neuroprotection through the PI3K/AKT/BAD/BCL-2 pathway.

In conclusion, the present study demonstrates that indirubin alleviates retinal neurodegeneration induced by the ONC injury and protects RGCs from oxidative damage. Mechanically, indirubin plays its neuroprotection through the PI3K/AKT/BAD/BCL-2 signaling pathway. Therefore, the present study provides new insights into potential therapeutic Chinese medicines for retinal neurodegenerative diseases.
